# Association of Low BMI, Elevated Model for End-Stage Liver Disease Score, and Poor Functional Status With Increased 30-Day Readmission After Orthotopic Liver Transplant: A Retrospective Cohort Study

**DOI:** 10.7759/cureus.81915

**Published:** 2025-04-08

**Authors:** Justin Lok, John Paliakkara, Catherine O'Leary, Ruston Keller, Alexxis Gutierrez, Aniketh Naidu, Raymond I Okeke, Mustafa Nazzal

**Affiliations:** 1 General Surgery, Saint Louis University School of Medicine, St. Louis, USA; 2 Family Medicine, Upstate University Hospital, Syracuse, USA; 3 Medicine, Saint Louis University School of Medicine, St. Louis, USA; 4 Surgery, Saint Louis University School of Medicine, St. Louis, USA; 5 General Surgery, SSM Health Saint Louis University Hospital, St. Louis, USA; 6 Surgery, Saint Louis University Hospital, St. Louis, USA

**Keywords:** 30-day readmission, body mass index (bmi), meld scores, orthotopic liver transplantation, transplant recipient

## Abstract

Background

Liver transplantation is the ultimate treatment for end-stage liver disease. However, post-transplant management is very complex, with the need for meticulous immunosuppression regimens and multidisciplinary coordinated specialist care. This can be complicated by a higher risk of readmissions after transplant. Readmission rates are now being used as a measure of a facility's efficacy post-transplant. In this study, we investigated recipient characteristics that may place recipients at higher risk for 30-day readmission.

Method

Chi-square and independent t-test analyses of six variables were performed accordingly on liver transplant recipient data extracted from the Standard Transplant Analysis and Research (STAR) data. The variables were Model for End-Stage Liver Disease (MELD) score, body mass index (BMI), age, diabetes status, hepatitis C status, and functional status. The association between these variables and 30-day readmission rates was investigated.

Results

We observed six recipient risk factors, including elevated MELD score, positive diabetes mellitus status, positive hepatitis C status, and lower functional status, which increase hospitalization post-transplant. Of the six examined characteristics, lower BMI, elevated MELD score, and lower functional status were significantly associated with 30-day readmission. The t values and P values were as follows: t(38,180) = 4.080, P = 4.514E-05 for BMI; t(38,180) = 4.080, P = 4.514E-05 for MELD score; and t(38,180) = 2.729, P = 6.356E-03 for functional status.

Conclusion

Our study shows that liver transplant recipients with lower BMI, higher average MELD score, and lower functional status can be identified as high-risk recipients for readmission within 30 days after liver transplant. These findings might help transplant centers anticipate higher complication rates and possibly implement better nutritional optimization prior to transplant and closer follow-up after transplant. Further research could identify specific thresholds for these characteristics that are associated with significantly worse outcomes.

## Introduction

Measuring recipient readmission rates has become an important quality metric for hospital efficacy [1]. Despite readmissions being a means to intervene on recipients with emergency complications, there are both economic and patient care incentives to minimize readmission rates [2]. Economically, there are wasted expenses due to mismanaged procedures, compounded by the Affordable Care Act reducing reimbursements for facilities with high readmission rates [3]. In terms of patient care, Zeidan et al. reported that 30-day readmissions following liver transplant have been independently associated with 90-day mortality, where the mortality rate rose from 9.8% to 26.8% in recipients who were readmitted to the hospital [4]. This makes it imperative that there is a concerted effort to minimize readmissions after hospital care.

Liver transplantation (LT) is an important part of the medical care arsenal, as it is the ultimate treatment for end-stage liver disease [5]. This condition is often caused by alcoholic cirrhosis, metabolic dysfunction-associated steatotic liver disease (MASLD), and hepatitis virus-associated liver disease [6]. As of 2023, alcoholic liver disease accounts for 40% of liver transplants, followed by MASLD and hepatocellular carcinoma [7]. LT shows a drastic improvement in the health and life expectancy of patients with liver disease, extending the two-year life expectancy of patients requiring transplant to ten years if transplantation is received [3]. Despite the high success rates and the ubiquity of LT, with 9,400 procedures performed in 2023 [8], a 2025 Milliman Research Report estimated that the billed charge per liver transplant in 2025 will be $1,017,800 [9]. Fortunately, for many transplant recipients, these procedures are covered by Medicare and Medicaid [10]. However, this means that medical errors and poor management of liver transplants ultimately become the financial burden of taxpayers [3,4,11].

Readmission rates may reflect the transplant center’s post-transplant management, recipient self-management, community resources, and co-morbidities. Post-transplant management is difficult for recipients because it requires careful immunosuppression, multidisciplinary coordinated specialist care, and the ability to manage complex regimens. There may also be recipient characteristics that place patients at higher risk for readmissions [5,12]. This study reviewed a large national dataset organized by the Organ Procurement and Transplantation Network to assess pre-transplant recipient characteristics that have been suggested to worsen liver transplant outcomes. These include recipient age [13], body mass index (BMI) [14], Model for End-Stage Liver Disease (MELD) score [15], diabetes mellitus status [16], hepatitis C virus (HCV) status [3], and functional status [17].

## Materials and methods

Standard Transplant Analysis and Research data

The data for this study were extracted from the Standard Transplant Analysis and Research (STAR) dataset, a publicly available, de-identified database containing detailed information on liver transplant recipients in the United States. The study focused on adult recipients aged 18 years or older who underwent LT. Pediatric recipients and those with incomplete data were excluded. Patients without a recorded value for any one of the analyzed variables were excluded. For example, a patient with a recorded diabetes status but no known or recorded HCV status would be excluded, ensuring that analyses for each variable were conducted within the same population. The variables analyzed included recipient age, BMI, MELD 3.0 [18] lab score at transplant (calculated from several necessary variables; see GitHub repository for exact names), presence of diabetes at registration, hepatitis C serostatus, and functional status at registration.

To assess post-transplant readmissions, hospitalization status and follow-up dates were extracted from the LIVER_FOLLOWUP_DATA table using the variables HOSP, which indicates whether a patient was hospitalized within the follow-up period, and PXSTATDATE, which provides the date of the follow-up assessment. Readmission was defined as any documented follow-up hospitalization occurring within 30 days after the transplant discharge date, which was determined using the DISCHARGE_DATE variable. Recipients undergoing re-transplantation were treated as independent cases and were recorded as separate transplant events. The STAR dataset does not contain a dedicated variable for 30-day readmission, but the difference in days between the discharge date and the follow-up date can be accurately calculated using standard programming techniques and widely accessible libraries.

Data management and statistical analyses were performed using a combination of Python (Python Software Foundation, Beaverton, Oregon), Java (Oracle Corporation, Austin, Texas), and Microsoft Excel (Microsoft Corporation, Redmond, Washington). Python and Java were primarily employed for data extraction, cleaning, compression, and transformation, while Microsoft Excel was used for most statistical calculations, including independent t-tests and chi-square analyses. All scripts developed for this study are publicly available in a GitHub repository (https://github.com/BharatBlade/LiverTransplantReadmissions) under an MIT License to ensure transparency and reproducibility. The repository includes raw and processed datasets, statistical analysis scripts, and Microsoft Excel files containing t-test and chi-square calculations. For specifics regarding statistical analyses, readers are referred to the relevant subsections for each variable. Due to differences in variable types (continuous versus categorical), not all variables underwent both t-test and chi-square analyses.

Missing values were assessed, and variables with excessive missing data were excluded to minimize bias. After these exclusions, 38,396 recipients with reliable data remained in the dataset. There were 214 recorded hospitalized recipients, and 38,182 fell into categories outside of hospitalized. To evaluate post-transplant readmission, recipients were stratified into two groups based on hospitalization status within 30 days of transplantation. Recipients readmitted during this period were classified as hospitalized, whereas those who were not readmitted were classified as non-hospitalized. For subgroup analysis, recipients were further categorized based on MELD score, BMI, age, diabetes status, HCV status, and functional status. In this study, integer notation will be used to define inclusivity (“[” and “]”) and exclusivity (“(” and “)”) of ranges of MELD scores and BMI.

All statistical analyses were conducted with an alpha value of 0.05 to determine significance. Results were interpreted in the context of prior literature, with findings assessed for clinical relevance in predicting early post-transplant readmission. This study utilized de-identified, publicly available data, and therefore, no Institutional Review Board (IRB) approval was required.

MELD score

Due to the MELD score’s ability to predict outcomes of patients on the transplant waitlist, MELD scores may be useful in predicting long-term recipient outcomes as well [19]. The MELD score calculated was averaged separately for hospitalized and non-hospitalized recipients, and differences between these groups were assessed using two-sample, independent t-tests with a significance level of 0.05. To explore the relationship between MELD scores and readmission rates, MELD scores were further stratified into four categories: 6≤MELD<15, 15≤MELD<21, 21≤MELD<28, and 28≤MELD≤40, based on prior literature investigating the impact of MELD score on resource utilization. A chi-square test was performed to determine whether there was a significant difference between the observed number of hospitalized recipients within each MELD category and the expected number based on population distribution.

Body mass index

BMI was similarly analyzed by comparing the mean BMI of hospitalized and non-hospitalized recipients on the day of transplant using two-sample, independent t-tests with a significance level of 0.05. To further investigate whether specific BMI ranges were associated with differences in hospitalization rates, BMI was categorized into six predefined groups: 0≤BMI<18.5, 18.5≤BMI<25, 25≤BMI<30, 30≤BMI<35, 35≤BMI<40, and 40≤BMI<50. A chi-square test was then used to assess whether a significant difference existed in hospitalization rates across these BMI categories.

Functional status

Previous literature showed that moderate to severe functional status was associated with worsened long-term liver transplant outcomes. There was an increased transplant length of stay, a greater requirement for ventilator support, and increased one-year post-transplant hospitalization and mortality [17,20]. Functional status was measured using chi-square analysis and two-sample, independent t-test analysis. Patients who were hospitalized and non-hospitalized at 30 days were stratified according to the Karnofsky Performance Scale (KPS) Index for the chi-square analysis. Recipients were categorized based on functional status at registration and stratified into hospitalized and non-hospitalized groups. A chi-square test was performed to determine whether there was a statistically significant difference in hospitalization rates across different levels of functional status. The quantitative differences in functional status between the hospitalized and non-hospitalized groups were assessed using two-sample, independent t-tests with a significance level of 0.05.

Age

The percentage of patients 65 years or older receiving liver transplants in the United States has increased from 14.4% to 29% through the years of 2010 to 2020 [10]. The mean age of hospitalized and non-hospitalized recipients was compared using a two-sample, independent t-test to determine whether age was associated with an increased risk of readmission.

 Diabetes mellitus

The impact of diabetes mellitus on liver transplant outcomes is inconsistent. This analysis included patients with diabetes mellitus, whether they were type 1 diabetic or type 2 diabetic. The literature describes both negative and neutral outcomes [21-23]. The impact of pre-transplant diabetes on readmission was evaluated by categorizing recipients based on diabetes status at the time of registration, including type 1 and type 2 diabetes, as well as cases where diabetes was documented without a specified type. Chi-square tests were performed to assess whether there was a significant association between pre-transplant diabetes and hospitalization status.

Hepatitis C

With the introduction of direct-acting antivirals (DAAs), there has been a decreased need for LT secondary to hepatitis C. Increased patient and graft survival have also been observed in patients who received DAA therapy for HCV [24]. Although HCV prevalence continues to decline, there is still an 8.3% prevalence of HCV-related LT at the time of waitlist registration [25]. Recipients were classified based on HCV serostatus at the time of transplant, and a chi-square test was conducted to determine whether a significant difference existed in hospitalization rates between HCV-positive and HCV-negative recipients.

## Results

 Model for End-Stage Liver Disease

Table 1 compares the average MELD score in hospitalized and non-hospitalized recipients. The average MELD score in hospitalized recipients was significantly higher than that of non-hospitalized recipients: t(38,180) = 4.080, P = 4.514E-05.

**Table 1:** Average Hospitalization vs Non-hospitalization *Values are significant. MELD: Model for End-Stage Liver Disease, BMI: body mass index, KPS: Karnofsky Performance Scale.

Recipient Characteristics	Hospitalized, Average	Non-hospitalized, Average	P-value
MELD*	25.50	23.03	4.54E-05
BMI (kg/m^2^)*	27.76	28.61	3.66E-02
Functional status (KPS)*	50.42	54.87	6.36E-03
Age (years)	53.80	54.35	0.500

Table 2 stratified the MELD scores into smaller groups: 6≤MELD<15, 15≤MELD<21, 21≤MELD<28, and 28≤MELD≤40. When stratified into smaller groups, the MELD score showed a general trend of increased percent hospitalization with elevated MELD scores across all time frames (Table 2). Chi-square analysis (X² = 16.228, df = 3, P = 1.02E-03) showed that there was a significant association between MELD score category and hospitalization status. The percentage of hospitalized recipients doubled when comparing 6≤MELD<15 to 28≤MELD≤40.

**Table 2:** Recipient Hospitalization and Average Status per MELD Score H: hospitalized; HA: hospitalized average; NH: non-hospitalized; NHA: non-hospitalized, average; E: expected; MELD: Model for End-Stage Liver Disease. *Values are significant.

MELD	HA	NHA	T-test P-value	H	NH	%	X^2^ P-value
6 ≤ MELD < 15	11.111	10.411	1.192E-01	27	8,537	0.32%	1.018E-03
15 ≤ MELD < 21	17.863	17.613	3.938E-01	39	8,150	0.48%	
21 ≤ MELD < 28	24.094	23.871	4.070E-01	60	8,883	0.67%	
28 ≤ MELD ≤ 40	34.267	34.482	6.117E-01	88	12,612	0.69%	
All	25.504	23.031	4.514E-05	214	38,182	0.56%	

Body mass index

Table 1 provides numerical analysis of the average BMI of hospitalized versus non-hospitalized recipients. It shows a statistically significant difference in BMI between hospitalized and non-hospitalized recipients, t(38,180) = 2.090, P = 3.665E-02.

Table 3 further analyzes the relationship between BMI averages of hospitalized and non-hospitalized recipients and the relationship specific BMI ranges had on post-transplant hospitalization. Recipients were stratified into BMI groups of 0-18.5 kg/m², 18.5-25 kg/m², 25-30 kg/m², 30-35 kg/m², 35-40 kg/m², and 40-50 kg/m². There was no significant difference between BMI categories in hospitalized and non-hospitalized recipients, as confirmed by chi-square analysis. Nor was there a significant association within specific BMI ranges and elevated hospitalization rates, as confirmed by t-test analysis for each BMI category. While individual categories had relatively similar rates of readmission, recipients in the underweight and healthy BMI ranges unexpectedly showed a technically higher rate of readmission, as indicated by the percentage of readmission.

**Table 3:** Recipient Hospitalization and Average Status per BMI H: hospitalized; HA: hospitalized, average; NH: non-hospitalized; NHA: non-hospitalized average; E: expected.

BMI (kg/m^2^)	HA (kg/m^2^)	NHA (kg/m^2^)	T-test P-values	H	NH	%	X^2^ P-value
0 ≤ BMI < 18.5	16.346	17.411	4.834E-01	3	499	0.60%	2.356E-01
18.5 ≤ BMI < 25	22.250	22.644	8.994E-02	75	10,358	0.72%	
25 ≤ BMI < 30	27.290	27.434	8.007E-01	67	13,517	0.49%	
30 ≤ BMI < 35	32.062	32.220	6.304E-01	42	8,598	0.49%	
35 ≤ BMI < 40	36.845	37.109	4.728E-01	20	3,816	0.52%	
40-50	44.390	42.539	5.182E-01	7	1,344	0.52%	
All	27.141	27.682	3.665E-02	214	38,132	0.56%	

Functional status

Table 4 compares recipients’ hospitalization status based on pre-transplant functional status (KPS). Differences in functional status were not initially statistically associated with increased hospitalizations, P = 1.37E-01, based on chi-square analysis. However, t-test analysis in Table 1 showed statistical significance, t(38,180) = 2.729, P = 6.356E-03, with lower functional status being associated with a higher rate of readmissions.

**Table 4:** Recipient Hospitalization Status per Functional Status

Functional Status	Hospitalized	Non-hospitalized	X^2^ P-value
10	11	1,466	1.369E-01
20	35	4,969	
30	20	2,815	
40	23	3,758	
50	26	4,788	
60	28	5,031	
70	39	6,366	
80	16	5,844	
90	13	2,269	
100	3	876	
Total	214	38,182	

Age

Table 1 provides numerical analysis of the average age of hospitalized versus non-hospitalized recipients. There was no statistical difference between the average ages of hospitalized and non-hospitalized recipients. The mean ages were 53.804 years and 54.347 years, respectively, t(38,180) = 0.675, P = 4.996E-01.

Diabetes

Table 5 compares the hospitalization status of recipients with positive and negative diabetes status. Positive diabetes status prior to transplant was not significantly associated with increased hospitalization, X² = 0.526, df = 1, P = 4.68E-01.

**Table 5:** Recipient Hospitalization vs Non-hospitalization per Diabetes Status and HCV Status HCV: hepatitis C virus.

Recipient Characteristics	Hospitalized	Non-hospitalized	P-value
Diabetes	61	10,045	4.681E-01
No diabetes	153	28,137	
HCV	79	13,847	8.441E-01
No HCV	135	24,335	

 Hepatitis C virus

Table 5 compares the hospitalization status of recipients with and without HCV. Positive HCV status did not show a significant increase in hospitalization, X² = 0.039, df = 1, P = 8.44E-01.

## Discussion

LT is the definitive treatment for end-stage liver disease. These procedures are performed on chronically debilitated patients with multiple comorbidities and thus represent a nidus for readmissions, which incur increased cost, resource utilization, and worse outcomes for post-transplant liver recipients [15,26]. This project used STAR data to assess the impact of MELD score, BMI, age of recipient at transplant, pre-transplant diabetes, pre-transplant HCV, and functional status on post-transplant hospitalization.

Results from this analysis showed that an elevated MELD score was significantly associated with 30-day readmissions. The average MELD score between readmitted and non-readmitted recipients had a difference of 2.47. This increase is consistent with data from the Nationwide Readmission Database from 2010 to 2014, which determined that high MELD scores were predictive of 30-day readmissions post-LT [3]. The analysis also found an increase in the proportion of hospitalizations in higher MELD score categories.

A secondary predictor of post-transplant readmissions was lower transplant BMI. This is in contrast to our prior assumption. Obesity is associated with cardiovascular disease, renal dysfunction, cancer, and pulmonary hypertension [27]. Due to these comorbidities, it was hypothesized that elevated BMI would play a role in the rate of re-hospitalization post-LT. This was not consistent with the comparison of average BMI between readmitted and non-readmitted recipients. Although there was statistical significance in the BMI data, the data showed that lower BMI was associated with elevated readmissions. It is suspected that this relationship is secondary to malnutrition or malabsorption as potential causes of this worsened outcome.

Moderate to severe functional status makes up 70% of liver transplant recipients [17]. Liver transplant recipients with worse functional status were reported to have inferior outcomes, presenting with increased length of stay, increased requirement for ventilator support, and increased one-year mortality and one-year graft failure [17,20]. Our review of STAR data showed a decrease in readmissions as functional status increased; however, this was not statistically significant via chi-square analysis (P > 0.05). There were no significant differences detected between these categorical groups. T-test analysis did show statistical significance, with lower functional status being associated with increased rates of readmission. Further research can assess the impact of prehabilitation on readmission rates, as current literature has shown that prehabilitation reduces postoperative complications in major abdominal surgeries, improves recipient frailty metrics, and decreases the length of stay in the ICU [28].

In parallel with the nationally increasing obesity rates, transplant recipients are undergoing procedures at older ages. This reflects a substantial change in the field of organ transplantation through improvements in safety and postoperative care of liver transplants for older recipients, making the procedure more accessible [29]. From these advancements, the impetus for liver transplant has shifted from recipients requiring transplantation due to hepatitis virus infection to transplantation due to MASLD or HCC [30]. Per this analysis, increased age showed little statistical distinction in average age between readmitted and non-readmitted recipients (P = 0.933), indicating that age does not significantly impact short-term readmission after LT.

Current literature shows varying associations between diabetes mellitus and post-transplant readmissions [21,22]. It was hypothesized that pre-transplant diabetes would lead to an increased risk of hospitalization after liver transplant within 30 days. Recipients showed no significant relationship between re-hospitalization and pre-transplant diabetes (P = 4.68E-01).

Similarly, analysis of HCV showed no significant association between pre-transplant HCV status and readmissions (P = 8.44E-01), even though previous literature revealed that liver transplant recipients of advanced age with a history of HCV had elevated post-transplant hospitalization rates. Notably, there is a decreased need for LT secondary to HCV because of the increased efficacy of direct-acting antiviral agents [29].

There were several limitations to the study. First, the study primarily revolves around univariate analysis. One major aim of future research is to develop multivariate models and provide ROC analysis to identify and create causal relationships between these characteristics and liver transplant outcomes. The endpoint for this readmission study is to create a score to prospectively assess a patient’s risk of readmission prior to transplantation. Another limitation is the large sample size of the STAR data, which increases the likelihood of a type 1 error. Further analysis should determine whether electronic health records of local transplant data correlate with national data. More practical avenues of readmission research would include identifying the impact that increased postoperative clinical visits have on transplant recipient outcomes. Increased touchpoints between the recipient and the provider before and after transplant may help prevent complications from occurring prior to a need for readmission.

## Conclusions

LT is a safe and effective tool in the treatment of end-stage liver disease, extending the two-year life expectancy of recipients requiring transplant to 10 years. However, LT comes with a high risk for readmissions, which can be costly for the health care system. This project used STAR data to assess the relationship between recipient characteristics, including recipient age, body mass index, MELD score, diabetes mellitus status, hepatitis C status, and functional status, and hospitalization within 30 days after transplantation. This project identified lower BMI, elevated MELD score, and lower functional status as characteristics that were significantly associated with increased readmission post-transplant. These recipients would be considered at higher risk, requiring increased intervention in the first 30 days post-transplantation to ensure improved outcomes. These variables must be taken into consideration when creating a post-operative plan for the patients, as higher MELD scores, lower BMIs, and lower functional status may require more careful follow-up to minimize future readmissions. The readmission data can be further assessed using multivariate analysis. Eventually, this data can be used to create a score to prospectively assess recipients’ risk of readmission.
